# Regulatory Aspects of Sabin Type 2 Withdrawal From Trivalent Oral Poliovirus Vaccine: Process and Lessons Learned

**DOI:** 10.1093/infdis/jiw564

**Published:** 2017-06-30

**Authors:** Daniela Decina, Jacqueline Fournier-Caruana, Marina Takane, Razieh Ostad Ali Dehaghi, Roland Sutter

**Affiliations:** 1 Regulatory Systems Strengthening Team,; 2 Prequalification Team,; 3 Water, Sanitation and Hygiene, and; 4 Research, Policy and Containment, World Health Organization, Geneva, Switzerland

**Keywords:** lessons learned, regulatory, World Health Organization, Polio Switch, prequalification.

## Abstract

Withdrawal of type 2 oral poliovirus vaccine (OPV) in OPV-using countries required regulatory approval for use of inactivated poliovirus vaccine and bivalent OPV in routine immunization. Worldwide, a variety of mechanisms were used by member states, with some differences in approach observed between inactivated poliovirus vaccine and bivalent OPV. These included acceptance for use of World Health Organization (WHO) prequalified vaccines, registration and licensure pathways, participation in WHO-convened joint reviews of licensing dossiers, as well as pragmatic application of alternatively available mechanisms, when appropriate. Simple but effective tools were used to monitor progress and to record, authenticate, and share information. Essential to achievement of regulatory targets was ongoing communication with key stakeholders, including switch-country national regulatory authorities, vaccine manufacturers, partner organizations, and relevant units within WHO. Understanding of the regulatory environment gained through the OPV switch can be helpful in supporting further stages of the polio end game and other time-sensitive vaccine introduction programs.

In 2013, the Polio Eradication & Endgame Strategic Plan 2013–2018 [1] was developed by the Global Polio Eradication Initiative to capitalize on any new opportunity to end all polio disease. Objective 2 called for the globally synchronized withdrawal of oral poliovirus vaccine (OPV), beginning with cessation of the use of type 2 OPV by mid-2016. At the 68th World Health Assembly, in 2015, resolution 68.3 [2] was adopted, in which member states committed to the withdrawal of the type 2 component of trivalent OPV (tOPV) in April 2016. The resolution called for the expedited registration of bivalent OPV (bOPV), containing only poliovirus strains 1 and 3, for use in routine immunization (RI) programs. The bOPV would thereby replace tOPV, containing strains 1, 2, and 3, in routine use in all OPV-using countries. The substitution of tOPV with bOPV in a short, defined time window has been termed “the switch.” As a risk mitigation measure, resolution 68.3 also called for the introduction of inactivated poliovirus vaccine (IPV), optimally before the withdrawal of the type 2 component of OPV in April 2016. The resolution provided the political support for engaging countries in the regulatory efforts required by the switch timeline.

Introduction of new vaccines into national markets requires regulatory mechanisms, in compliance with statutes of the country. Successful implementation of the switch, as well as addition of 1 dose of IPV on top of bOPV in the RI schedule [3], thereby included regulatory elements to enable compliant vaccine introductions. Worldwide, various mechanisms can be used to permit the use of a vaccine either in routine or in exceptional circumstances. Collectively, these are often referred to as “regulatory pathways,” and they may or may not involve conventional product licensure. Therefore, throughout this work, the enabling mechanisms for vaccine introduction were termed “approval for use,” to capture all pathways leading to vaccine introduction.

Before the global switch window (defined as 17 April to 1 May 2016) [4], the total number of countries using tOPV in RI was 148, in addition to 7 territories. Use of IPV for RI was in place in 46 of 194 countries before resolution 68.3. These countries were categorized as “nonswitch countries” and were consequently out of the scope of the regulatory objectives. Another 22 countries were already users of IPV in some way, in addition to tOPV use. Therefore, the number of OPV-only countries required to achieve approval for use of IPV was 126 at the outset of the initiative.

All IPVs and bOPVs that were either procured through the United Nations Children’s Fund (UNICEF) or self-procured were licensed in their country of origin and prequalified by the World Health Organization (WHO). The 2 prequalified IPVs, manufactured by Bilthoven Biologicals and Sanofi Pasteur were licensed by the national regulatory authorities (NRAs) of the Netherlands and France, respectively. The 6 manufacturers of prequalified bOPV [5, 6] were Bharat Biotech International (India), GlaxoSmithKline Biologicals (Belgium), Haffkine Bio Pharmaceutical (India), PT Bio Farma (Indonesia), Sanofi Pasteur (France), and Serum Institute of India (India), licensed by their respective NRAs.

A number of OPV switch countries were self-producers of bOPV. These were Brazil, China, India, Indonesia, Iran, Mexico, the Russian Federation, and Vietnam. Manufacturers in all self-producing countries were required to obtain an authorization from the regulatory authority of their country to market the vaccine (a marketing authorization) before use.

## REGULATORY GOALS OF THE SWITCH

The aim of the introduction of bOPV and IPV was reflected in the regulatory goals and support activities. The regulatory goal in support of the tOPV-to-bOPV switch was the approval for RI use of ≥1 source of bOPV before the switch window, with approval of >1 source preferred. The timeline for bOPV introduction was driven by the switch window. Data tracking bOPV approvals were collected in the year leading up to the switch.

Although the administration of ≥1 dose of IPV in RI was optimally to be introduced to OPV-using countries before the switch [2], the objectives were modified in response to global IPV shortages. The regulatory goal for IPV introduction was, therefore, only time-bound to the switch window for high-risk countries and was defined for all countries as no delay in access to IPV based on a regulatory approval for use. Tracking of IPV approvals began in the first quarter of 2014 and is ongoing.

## ROLE OF WHO

Throughout the project, the role of WHO in regulatory support was to (1) gather existing data about the regulatory environment learned from previous experiences—for example, introductions of monovalent OPV types 1 and 3 (mOPV1 and mOPV3), bOPV (in a campaign setting), and meningitis vaccine; (2) facilitate communication and bridge stakeholders, including NRAs, manufacturers, procurement agencies, partner organizations, WHO regional and country offices, and the concerned teams at WHO headquarters (Polio Eradication, Expanded Programme on Immunization, Essential Medicines and Health Products); (3) track progress of the achievement of approvals for use; (4) identify areas of regulatory focus or concern that could jeopardize on-time switch; (5) conduct workshops facilitating review of IPV licensing dossiers; (6) provide scientific background information packages for IPV and bOPV to help countries in their regulatory decision; and (7) update and retain the regulatory pathways data collected for bOPV and IPV to facilitate emergency vaccine introduction in the future, when and where needed.

## PROGRESSION OF THE APPROVAL FOR USE OF bOPV AND IPV

**Figure 1.**
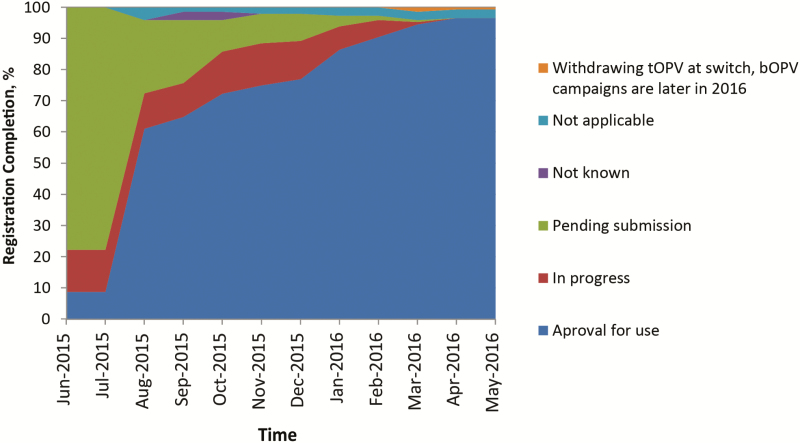
Bivalent oral poliovirus vaccine (bOPV) registration status by month in the year leading to the switch. Abbreviation: tOPV, trivalent oral poliovirus vaccine.

**Figure 2.**
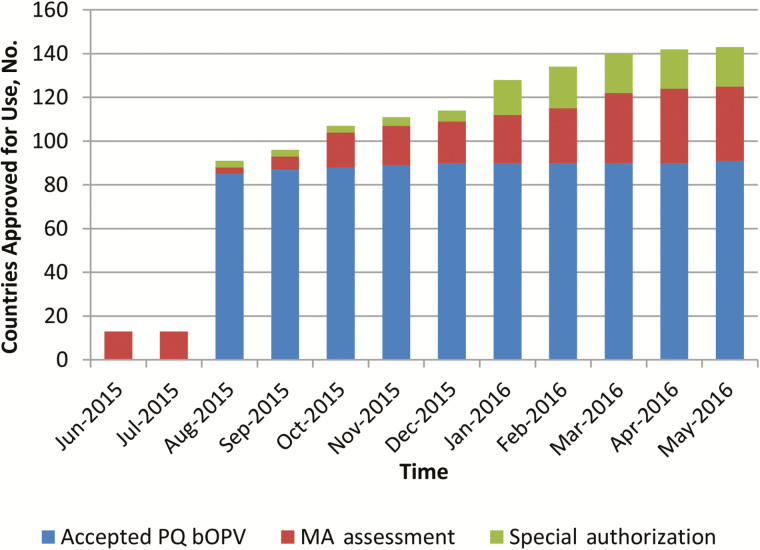
Bivalent oral poliovirus vaccine (bOPV) approval for use mechanism by month in the year leading to the switch. Abbreviations: MA, marketing authorization; PQ, prequalified.

When bOPV was introduced in December 2009, its use was limited to supplementary immunization activities (SIAs), and tOPV remained the vaccine for RI. The introduction of bOPV after withdrawal of tOPV required a label change from SIA to RI, approved by the NRAs of record and WHO; and this variation was completed in 2015. Consequently, tracking of bOPV registration status only began in June 2015. Figure 1 shows that <10% of switch countries had an approved bOPV, 10% had reviews in progress, and almost 80% were pending initiation of a regulatory activity. The sharp increase in approval for use in August 2015 marked the completion of the prequalification of the first bOPVs for RI. For four switch countries, information on their registration requirements and status were temporarily unclear (not known). Four countries elected to discontinue their use of OPV, while maintaining their IPV immunization schedules, and were classified as not applicable for bOPV approval. One country withdrew tOPV at the time of the switch but would not require bOPV until late 2016. Marketing authorization was obtained in August 2016. Vaccine access through acceptance of prequalified vaccine was the regulatory pathway for the majority of OPV-using countries (Figure 2). A marked increase in use of special authorization pathways was noted 1 quarter before the switch window. These could include waivers, tenders, or other expedited approval mechanisms.

**Figure 3.**
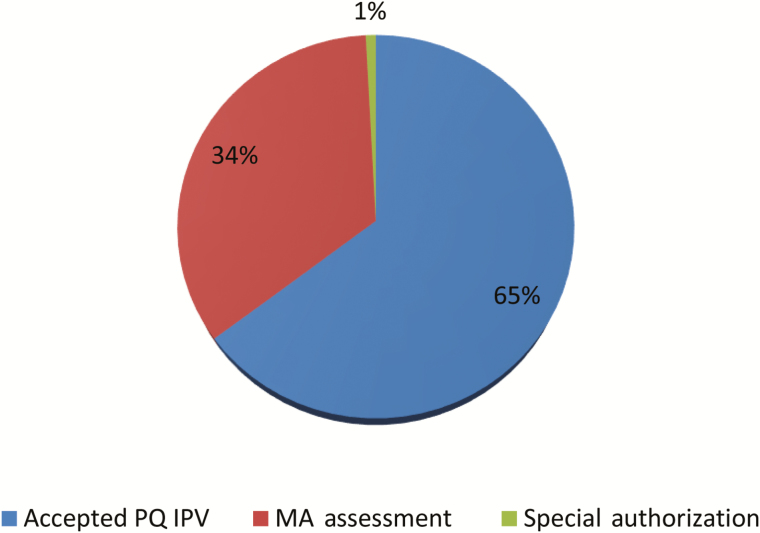
Inactivated poliovirus vaccine (IPV) approval for use mechanism in countries introduced at the time of the switch. Abbreviations: MA, marketing authorization; PQ, prequalified.

The goal to obtain an approval for use of IPV before introduction was achieved in all cases, with no IPV introductions delayed because of regulatory issues. Directly after the switch, 7 of 126 countries remained to complete a regulatory pathway for IPV use. Each was expected to achieve approval before their IPV introduction. The pathways used for IPV introduction were the same as those for bOPV introduction and in similar proportion (Figure 3). Notable is the smaller number of countries that implemented a special authorization, which may reflect the earlier start of regulatory activities and deadlines not tied to the switch window.

## LESSONS LEARNED

Eight key regulatory lessons were identified and are outlined below.

### Lesson 1: Understand the Regulatory Environment

A first step toward achieving the regulatory goals was understanding the baseline regulatory landscape. Switch countries were classified by anticipated regulatory pathway to understand the workload and to facilitate the tracking of achievement of approval for use. Pathways included acceptance of prequalified vaccine, full assessment of a dossier, and ability to use the WHO collaborative procedure, a procedure by which a regulatory authority can take advantage of scientific assessment work conducted by WHO when reviewing a marketing application for a new vaccine [7]. Countries whose anticipated pathway was not clear were flagged for follow-up. The baseline was established using previous knowledge within WHO headquarters and regions, manufacturers, and UNICEF. The baseline data were verified or updated in collaboration with WHO regional offices and UNICEF Supply Division. Differences at baseline were resolved by assuming the most conservative pathway until verified otherwise. Routine and ongoing communications with stakeholders were used to update knowledge. Three pathways to approval for use were identified: acceptance of prequalified vaccines, assessment of marketing applications, and special authorizations.

#### Acceptance of WHO-Prequalified Vaccines

Acceptance of prequalified vaccines without extra regulatory evaluation continues to be the most used pathway for vaccine access for the public market, with reliance on the WHO vaccines prequalification program for the quality and clinical assessment of the product, which includes review of the dossier, sample testing, and site inspection. The prequalification program was an important contributor to the goal of having >1 approved bOPV source, because each additional prequalified vaccine increased supply planning options for the country and the procurement agency.

The principle procurement sources were UNICEF and the Pan American Health Organization Revolving Fund, but vaccines could also be directly from suppliers by self-procuring countries (30% of countries). Prequalification significantly enables access to vaccine in emergencies, including mOPV2 in outbreak response. Countries using special authorizations to achieve timely bOPV introduction also relied on prequalified vaccines to accept products of assured quality in lieu of, or concurrent with, their own technical reviews. Establishment or strengthening of technical review capabilities by NRAs adds to, but should not entirely replace, pathways accepting prequalified products. Strengthened regulatory systems include the flexibility to respond in a timely manner to public health needs.

#### Marketing Application Assessment

Marketing application assessment was the second most used regulatory pathway both for IPV and bOPV, requiring submission of a technical dossier to the NRA of the country, with the outcome of a licensure or registration of the vaccine. Such assessments may have included the request for facility inspections or samples testing.

Approximately one-third of countries using an assessment pathway were documented as having a registered bOPV for SIAs. In addition, bOPV could be considered tOPV without the type 2 strain component. This provided the opportunity to consider a bOPV application as a variation dossier rather than a new vaccine. In this project, bOPVs granted licensure were not differentiated by whether they were assessed as new applications or variations. However, anecdotal evidence suggested bOPV licensures generally proceeded as new applications, and there may be an opportunity to compare efficiency of new versus variation applications in future vaccine introduction initiatives.

A number of countries were part of a regulatory network in which licensure in one country could facilitate approval in other countries in the network; however, it is not known to what extent this mechanism was used to achieve licensure for poliovirus vaccines. For example, bOPV approval in Gulf Cooperation Council countries ranged from July 2015 to January 2016, but an approval cascade based on a networked approach was not clear.

Approximately 10% of switch countries were categorized at baseline as users of the WHO collaborative review procedure [7]. This is a procedure for sharing information on the scientific assessment work conducted by the WHO Prequalification Team. Although the procedure was primarily used for drugs and extended to vaccines only in mid-2015, its use is expected to increase for future vaccine licensures.

#### Special Authorizations

Expedited approval mechanisms were grouped under the term “special authorization,” which included waivers and tenders. Where a special authorization was applied, it could be without limitation or may have been a temporary time-bound or quantity-specific authorization. As such, special authorizations should be seen as bridging strategy and flagged for follow-up to identify instances where parallel licensure pathways are ongoing and require completion for supply continuity.

It is valuable to understand who the decision maker is within the country’s health system authorizing the special approval and whether that body is the same as the one issuing required importation documentation. Vaccine introduction can be hampered when special authorizations are not coordinated between the Ministry of Health and the NRA.

### Lesson 2: Manage Regulatory Risk

Regulatory risks to the success of the switch were identified, and strategies implemented for mitigation. Tracking of approvals for use in real time, in particular as the switch window approached, was essential to directing regulatory efforts for resolution of impediments. This was accomplished through regular and frequent communication with key stakeholders, consisting of the internal team, WHO regional offices, UNICEF, and vaccine manufacturers, including teleconferences and face-to-face meetings. Progress, identified risk areas, and strategies for mitigation were shared. Communications and meetings with NRAs were added as needed, to facilitate resolution of country-specific approval risks, either directly or through WHO regional focal points.

Joint review workshops were organized to expedite IPV licensure. Three regional workshops were held, in Africa, the Eastern Mediterranean region and Southeast Asia, with 19 participating countries. The workshops brought NRAs together in joint evaluation of the dossiers, with participation of representatives of the producer country NRAs and manufacturers. The goal was to achieve regulatory approvals within 3 months of the workshops; however, in reality, approvals generally significantly exceeded the targeted time frame. Contributing factors may have included submission queue times, changes in personnel involved, differences between the prereview and final packages, and country-specific administrative requirements. Positive feedback included the opportunities for discussion, collaboration, and knowledge exchange. However, it was concluded that this type of workshop alone is not sufficient to expedite product registration.

Based on previous experience gained with introduction of mOPV1, mOPV2, and mOPV3, regulatory information packages were prepared and shared with the NRAs of switch countries. This scientific background provided consolidated information on safety and immunogenicity of bOPV and IPV gathered by WHO and partners through clinical trials conducted in different settings. It was intended to assist NRAs in their decision-making process and to expedite approval for use.

### Lesson 3: Focus on Challenge Areas

A number of challenge areas were identified but were not unique to this program and are therefore highlighted for similar projects. Among the stakeholders of UNICEF, manufacturers, and WHO, it has been recognized that a diversity of country-specific requirements exist that can require tailoring of individual dossiers. This can lead to delays in filing, screening, and review of applications and assembly of import documentation. Efficiency can be gained in 2 ways. First, document regulatory requirements, with continuous updating as requirements evolve, and share across stakeholders, to reduce delays in preparing documents or delays from requests for additional information. Second, foster convergence and harmonization of requirements globally. This is a longer-term approach that can be complicated by the structure of legal frameworks that have unduly encompassed a high degree of technical detail at the level of law. It can be assisted by increasing adoption of Good Regulatory Practices to ensure that appropriate flexibilities are built into regulatory frameworks.

Sample testing and inspection of manufacturing facilities can be requirements of the full assessment of a marketing application for a new vaccine and can be driven by legislative requirements. Confirmation of product quality and Good Manufacturing Practice compliance is imperative but can lead to extensive retesting and reinspection, with limited incremental gain in quality assurance. In this project, retesting of prequalified vaccines was discouraged, and requests for inspections were minimized by advocating recognition of, or reliance on, the work done by WHO and the NRAs of producer countries.

Some self-procuring countries experienced difficulties in bOPV sourcing. Where the countries required a dossier review, it was important that sourcing was aligned with dossiers submitted or approved. Identification and communication of gaps enabled a crosscutting approach to timely resolution.

### Lesson 4: Expect Specific Challenges for Countries Self-Producing bOPV

Specific attention was directed to 7 countries planning to use a local producer for their access to vaccine. One success factor was to begin dialogues with self-producers as early as possible. The regulatory messaging for global switch readiness was shared by the Global Polio Eradication Initiative as early as September 2013. Note that registration requirements in self-producing countries can be more extensive, including the completion of local clinical trials. Introductory and follow-on face-to-face meetings organized by WHO between the local manufacturer and the NRA facilitated dialogue to ensure on-time registration of the vaccines. Regulatory requirements were discussed to clarify understandings and identify hurdles, which included approaches to meeting clinical trial data requirements.

### Lesson 5: Communicate and Collaborate With Stakeholders

Working collaboratively with stakeholders, with frequent communication and information-sharing was a major success factor. Such collaboration enabled timely progress tracking and a multipronged approach to problem solving regulatory hurdles. Status update meetings held at regular intervals with manufacturers were critical to assuring that WHO’s regulatory goals were achieved according to targets. These were held with increasing frequency in the last quarter before the switch and included face-to-face meetings, teleconferences, and email correspondence.

Meetings with regional offices and partner organizations were used to inform strategy, share progress, and troubleshoot barriers to vaccine introductions. In these fora, the focus areas—such as countries whose regulatory pathways were not clear, those at risk for not achieving on time approvals, and options for facilitation—were agreed on.

Collaboration with regional offices provided interpretations and context to country regulatory requirements and validated the status of approvals. Regional offices were the key points of contact with NRAs to obtain clarity on progress toward approval of vaccines and to facilitate resolution of issues. Headquarters representatives joined meetings with NRAs or health ministries to escalate advocacy as needed. Close collaboration and communication mechanisms also allowed rapid identification of issues and risks, as well as rapid response to these issues and sharing of solutions across stakeholders.

### Lesson 6: Track and Share Progress

Data management tools were essential to meet stakeholder requirements for up-to-date information on regulatory approval for use to ensure that the vaccine supply was not impeded. Several simple but effective tools were used to manage data. Spreadsheets were created to record, analyze, and share data for verification and monthly progress reporting. They were maintained on a SharePoint site with the version history function enabled, permitting data mining at time points throughout the collection history. Electronic tools facilitated timely routine reporting of progress and became a flexible means for rapid response to ad hoc queries, as well as data verification and synchronization across stakeholders. Regulatory data were funneled to dashboards and were mapped by the Immunization Management Group on the WHO Immunization, Vaccine and Biologicals website [8] for timely and transparent progress reporting and to maintain momentum toward goals.

### Lesson 7: Support Development of Pragmatic Regulatory Frameworks

The approval of vaccines for use in a country must occur through pathways within the country’s legal framework. However, the ability to respond in a timely manner to public health needs requires that the framework include pragmatic options. This is achieved by having a variety of regulatory pathways for adapting to time-sensitive situations. Our observations can benefit other vaccine introductions.

We noted in our polio data that of the 58 countries requiring dossier assessment, 30% used a special authorization pathway to access bOPV. Suitability of the vaccine was based on prequalification or assessment by the NRA of the producer. This demonstrated that flexibility for emergency response was present and is probably more prevalent than our data showed. In a number of large countries, provisions for emergency use are not yet in regulatory frameworks, highlighting areas for working with NRAs. Lack of an emergency pathway can have a large impact in situations such as deployment of the mOPV2 stockpile, wherein global licensure is not realistic.

The responsible and structured use of expedited regulatory approval mechanisms should not been seen as a shortcut from standard assessment mechanisms when vaccine suitability for use is supported by trusted sources. As regulators become better resourced and experienced, it is important to balance increased capacity for product assessment with flexibility to respond to emergencies through recognition of, and reliance on, the work of others (trusted NRAs and WHO). Sample and inspection requirements should fit risks and product knowledge. Use of the WHO collaborative review procedure may be a means of supplementing knowledge to reduce the need to repeat work and aid best use of limited NRA resources.

### Lesson 8: Maintain Acquired Knowledge

A considerable amount of data on the regulatory environment was collected in support of IPV and bOPV introduction. Maintenance and expansion of this data have continuing value. Extracts from these data have already been used to help formulate regulatory strategy for mOPV2 outbreak response and preparedness work in other disease areas.

The regulatory landscape is continuously evolving, driving a need to regularly update documented knowledge in a timely manner and in a searchable repository. For example, we noted that a number of countries were on the cusp of transitioning from reliance on prequalification to instituting independent technical reviews. In one such case, bOPV shipments for the national switch day were accepted on the basis of prequalification, while the NRA advised that reorder quantities would fall under a new licensure requirement. Such examples underline the importance of continuously updating baseline knowledge of country requirements.

Simple tools are adequate when accessed by small and limited user groups. However, there is value in considering database platforms to maintain information in the long term, not only to provide timely current data to new programs but also to archive superseded data in order to capture the progress of larger regulatory initiatives, such as the WHO collaborative review procedure for vaccines, networking and harmonization initiatives of NRAs, and implementation of Good Regulatory Practices.

## CONCLUSIONS

The accomplishment of the regulatory goals in approval for use of bOPV and IPV was a prerequisite for the introduction of these vaccines and an important component in accomplishing the switch. Strong collaboration between stakeholders, advocacy for the use of the WHO collaborative review procedure established by WHO through workshops, and scientific support to the regulatory authorities and Ministries of Health enabled an achievement of this magnitude over a timeline that was aggressive by regulatory standards. A legacy of this work is an enhanced understanding of the regulatory environment to benefit future vaccine introductions.

## Notes

***Acknowledgments.*** We thank WHO regional and country offices for their tremendous contribution to the regulatory work that enabled a timely switch in all OPV-using countries. We wish to recognize the strong collaboration between the UNICEF Supply Division, the Pan American Health Organization Revolving Fund, manufacturers, NRAs, and WHO that contributed to this unprecedented success.

***Financial support.*** This work was supported by the Global Polio Eradication Initiative and Immunization Management Group.

***Supplement sponsorship.*** This work is part of a supplement coordinated by the Task Force for Global Health with funding provided by The Bill and Melinda Gates Foundation and the Centers for Disease Control and Prevention.

***Potential conflicts of interest.*** All authors: No reported conflicts.
